# A decline in inflammation is associated with less depressive symptoms after a dietary intervention in metabolic syndrome patients: a longitudinal study

**DOI:** 10.1186/1475-2891-13-36

**Published:** 2014-04-24

**Authors:** Aurora Perez-Cornago, Rocio de la Iglesia, Patricia Lopez-Legarrea, Itziar Abete, Santiago Navas-Carretero, Clara I Lacunza, Francisca Lahortiga, Miguel A Martinez-Gonzalez, J Alfredo Martinez, M Angeles Zulet

**Affiliations:** 1 Department of Nutrition, Food Science and Physiology, University of Navarra, Irunlarrea 1, Pamplona 31008, Spain; 2 Faculty of Health Science, Universidad Autonoma de Chile, Santiago, Chile; 3 Biodonostia Health Research Institute, Doctor Begiristain (no number), 20014, San Sebastian, Spain; 4 Carlos III Health Research Institute, CIBERobn, Physiopathology of Obesity and Nutrition, Madrid, Spain; 5 Department of Psychiatry and Medical Psychology, University Clinic of Navarra, Pio XII 36, Pamplona 31008, Spain; 6 Department of Preventive Medicine and Public Health, University of Navarra, Irunlarrea 1, Pamplona 31008, Spain

**Keywords:** Metabolic syndrome, Depression, Inflammation, Leptin, Hypocaloric diet, Adiposity

## Abstract

**Background:**

Metabolic syndrome (MetS) and depression have become two prevalent diseases worldwide, whose interaction needs further investigation. Dietary treatment for weight loss in patients with MetS may improve depressive manifestations, however, the precise interactive pathways remain uncertain. Therefore, the aim of this study was to examine the effects of a hypocaloric diet designed to reduce MetS features on self-perceived depression and the possible underlying factors.

**Methods:**

Sixty subjects (Age: 50 ± 1 y; BMI: 36.1 ± 0.6 kg/m^2^) with MetS were selected from the RESMENA study (control and intervention) after they completed the 6-months hypocaloric treatment and rated for depressive symptoms using the Beck Depression Inventory (BDI). Anthropometric and biochemical measurements including leptin, C-reactive protein (CRP) and insulin levels were evaluated.

**Results:**

Depressive symptoms decreased during the weight loss intervention, with no differences between both dietary groups (control group −4.2 ± 0.8 vs RESMENA group −3.2 ± 0.6, *P* = 0.490). The number of criteria of the MetS was higher among subjects with more somatic-related depressive symptoms at baseline (B = 1.032, *P*-trend = 0.017). After six months of dietary treatment, body weight decreased in all subjects (−8.7%; confidence interval (95% CI) = 7.0-9.7) and also self-perceived depression (−37.9%; 95% CI = 2.7-4.9), as well as circulating leptin (−20.1%; 95% CI = 1.8-6.8), CRP (−42.8%; 95% CI = 0.6-3.0) and insulin (−37.7%; 95% CI = 4.1-7.2) concentrations. The decrease in BDI was significantly associated with declines in body fat mass (B = 0.34, 95% CI = 0.11-0.56) and also with the decrease in leptin (B = 0.16, 95% CI = 0.04-0.28) and CRP (B = 0.24, 95% CI = 0.01-0.46) concentrations.

**Conclusions:**

The decrease in depressive manifestations after a weight loss intervention was related with adiposity, CRP and leptin in subjects with MetS.

**Trial registration:**

ClinicalTrials.gov: NCT01087086.

## Background

The metabolic Syndrome (MetS) is defined as a cluster of major cardiovascular risk factors including central obesity, glucose intolerance, hypertension and serum lipid disorders, whose prevalence is rapidly increasing worldwide [1,2]. Similarly, certain MetS features such as excessive adiposity, glucose intolerance and dyslipidemia, have been associated with depression [3], which is considered the fourth leading cause of disease burden in the world [4].

Because of the high prevalence and public health implications of both depression and MetS, the potential association between them has recently received much attention [5,6]. However, the exact interactive pathways between these diseases still remain uncertain, although they seem to be bidirectional and predisposed by both biochemical and behavioral mediators [6]. Depression involves dysregulation of the adrenocortical and autonomic nervous systems, which could increase MetS risk by favoring abdominal fat accumulation and insulin resistance [6]. Furthermore, subjects with MetS present increased levels of inflammatory cytokines and leptin resistance. Thus, C-reactive protein (CRP), a serum marker of systemic inflammation, has been a frequently investigated inflammatory marker in subjects with MetS [7]. Also, chronic inflammation could be involved in mood disorders, as a positive relationship between depression and CRP has been reported [8]. Regarding the association between depression and leptin, an adipokine mainly secreted by adipocytes with a key role in energy regulation, it seems that resistance to this hormone may contribute to higher depression rates in obese subjects [9].

Various strategies have been proposed to counteract MetS manifestations, including lifestyle (diet and exercise) modification and drug therapy based on antihypertensives, insulin sensitizers or therapies for dyslipidemia [10]. One of the most prescribed lifestyle changes is dietary treatment for weight loss, where the Mediterranean diet has been proven to be a useful tool to improve both MetS and depression symptoms [11,12]. Also, the psychological effects of weight loss approaches have been a matter of controversy, mainly regarding how changes in body weight correlate with depressive symptoms [13].

This research is based on a subsample of the RESMENA-S study [14-17], a randomized, controlled intervention study that aims to reduce MetS features using a hypocaloric diet during six months. In this article, we hypothesized that a hypocaloric treatment designed to reduce MetS features produces a positive effect on depressive symptoms, and we sought to explore the possible underlying mechanisms and interactions of this effect.

## Subjects and methods

### Subjects

A total of ninety-three subjects (52 M/41 F) with a body mass index (BMI) of 36.1 ± 0.6 kg/m^2^ aged 50 ± 1 years diagnosed with MetS according to the IDF cut-offs [18] started the weight loss treatment. The inclusion and exclusion criteria have been previously reported [14-17], but it should be pointed out that subjects following antidepressant treatment were excluded as well as those with past mood disorders, including eating disorders. Also, vitamin or mineral supplements were not allowed.

**Figure 1:**
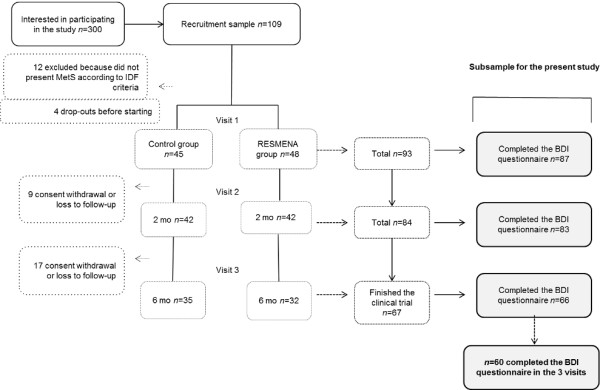
**Flowchart of the randomized and controlled dietary intervention for adults with MetS.** The 26 volunteers that dropped out did so for personal reasons not related to the study. Abbreviations: BDI, Beck Depression Inventory; IDF, International Diabetes Federation; MetS, Metabolic Syndrome.

After six months of weight loss intervention there were twenty-six dropouts due to loss to follow-up or consent withdrawal. Seven of the sixty-seven participants that finished the study did not complete all the Beck Depression Inventories (BDI). Therefore the present longitudinal study assessed the data from those 60 subjects (age: 50 ± 1 y.; 38 M/22 F), who completed the BDI in the three main visits (baseline, after two months and at the end of the study), as described in the study flowchart (Figure 1).

In the RESMENA study, the CONSORT 2010 guidelines [19] were followed, and all the volunteers signed written informed consent before participating in the intervention study. The study protocol was performed in accordance with the ethical guidelines of the Declaration of Helsinki, and was approved by the Research Ethics Committee of the University of Navarra (ref. 065/2009).

### Study protocol

The current study is based on a subsample of the RESMENA-S study, a randomized controlled intervention study aiming to improve clinical criteria and biomarkers associated with MetS through a dietary strategy for weight loss during six months. Briefly, participants were randomly assigned to follow one of the two energy-restricted diets, the control diet [20] or the RESMENA diet [14-17], both with the same energy restriction (−30% energy of the calculated requirements) by using the “random between 1 and 2” function in the Microsoft Office Excel 2003 software (Microsoft Iberica, Spain). The diets composition, as well as the different 48-h dietary records, were analyzed by the DIAL (Alce Ingenieria, Madrid, Spain) software and described elsewhere [16,17].

The volunteers were asked to maintain their usual physical activity, which was controlled by a 24-h physical activity questionnaire at the beginning and at the end of the study [21]. A psychological control using the BDI questionnaire was carried out at the main time points previously mentioned. More aspects of this intervention study have been previously detailed [14-17].

### Anthropometric and biochemical measurements

Anthropometric and body composition measurements were taken following standardized procedures previously described [15-17].

Serum glucose, total cholesterol, HDL-cholesterol, triglycerides and free fatty acids serum concentrations were measured in an autoanalyser Pentra C-200 (HORIBA ABX, Madrid, Spain) with specific kits. Insulin concentrations were determined by an enzyme-linked immunosorbent assay (ELISA) kit (Mercodia, Uppsala, Sweden) using an automated analyser system (Triturus, Grifols, Barcelona). Serum insulin resistance was estimated by the Homeostasis Model Assessment Index (HOMA-IR) calculated with the following formula: HOMA-IR = fasting glucose (mmol/L)xfasting insulin (mU/L)]/22.5 [22].

Plasma concentrations of CRP was assessed by an Immunodiagnostic AG kit (Bensheim, Germany) using an automated analyser system (Triturus, Grifols, Barcelona). Serum concentrations of leptin were measured using a RIA-based method (Diagnostic Products Corp., Los Angeles, CA).

### Assessment of depressive symptoms

Symptoms of depression were assessed at the main three time points of the study (baseline, after two months and at the end of the study) using the Spanish version of the BDI [23], which is considered a validated and reliable measure of depressive symptoms [24,25]. A score ≥ 10 reflects moderate depressive symptoms. The BDI questionnaire was divided into two subscales: the cognitive and the somatic symptom components. Question number 19 of the test, relating to weight loss, was discarded in some of the analyses given that losing weight is considered a depressive symptom, but our volunteers estimated it a positive aspect because they were enrolled in a weight loss treatment program [17].

### Statistical analyses

The sample size for the main study (the RESMENA study) was calculated based on previous findings [26], where the mean difference in the waist circumference was 4.3 cm and the standard deviation (SD) was 6.8 cm. To test the hypothesis of the present substudy and taking into account previous studies [27], a sample size of 29 subjects would be enough to obtain a statistically significant difference in the reduction of BDI score (2.2 ± 2.5 units), with an alpha error of 5% and a power of 90%.

The main results were summarized as mean ± SEM. Following published studies, the BDI score was analyzed as a continuous variable [9]. The Kolmogorow-Smirnov and the Shapiro Wilk test were used to analyze the normality of the measured variables. Analysis of covariance (ANCOVA) was used to assess changes between dietary treatments for the main dietary variables of the study, with sex and age as covariates. Changes in anthropometric and biochemical variables, as well as changes in the BDI questionnaire and physical activity level, were evaluated by repeated-measures ANOVA (three time points) or by using the nonparametric Friedman test when variables followed a non-normal distribution. Also, the effect of diet on these variables as well as the time-diet interaction was taken in consideration. A Bonferroni *post hoc* multiple comparison analysis was used when appropriate.

Tests of linear trend by increasing MetS criteria were conducted by assigning the mean value of BDI (somatic and cognitive questions separately) and modeling these values as a continuous variable. The changes in the cognitive and somatic questions (6 months – baseline) were assessed by ANCOVA with sex, age and dietary group as covariates. The association between changes (6 months – baseline) in BDI questionnaire and changes (6 months – baseline) total fat mass (kg) was analyzed using multivariable linear regression analysis adjusted by sex, age and dietary group. For the association between changes in CRP, leptin and insulin with changes in BDI score three models were constructed: Model 1 included sex, age and dietary group variables. Model 2 included model 1 additionally adjusted for changes in activity level, and model 3 consisted of model 1 plus changes in total fat mass (kg). All statistical analyses were carried out using SPSS 15.0 software for Windows (SPSS Ibérica, Madrid, Spain). Differences were considered statistically significant at *P <* 0.05.

## Results

The main dietary characteristics of the two prescribed diets were compared showing that, as expected, the RESMENA group consumed more protein than the Control group during the dietary treatment. Moreover, the RESMENA group showed a greater decrease in lipid intake, but lower intake of ω3 PUFA (Table 1). After six months of dietary treatment, subjects’ anthropometric and biochemical variables improved significantly, observing a mean weight loss of 8.4 ± 1.2 kg (*P <* 0.001). However, there were no differences (*P* ≥ 0.10) between both dietary intervention groups in neither anthropometric nor biochemical variables, as well as activity level of the volunteers. Also, both Control and RESMENA diets proved to be equally effective on improving the BDI score (Table 2). Therefore, as no significant differences between dietary groups were found in any of the variables analyzed in this study, henceforth the two groups were merged and analyzed together as a unique experimental group.

**Table 1:** Comparison of the two dietary treatments, control diet and RESMENA diet

	**Control group (n = 32)**		**RESMENA group (n = 28)**		***P *** **value**
**Dietary intake**	**Baseline**	**6 months**	**Baseline**	**6 months**	
Total energy intake, *Kcal/d*	2108 ± 69	1535 ± 54*	2279 ± 99	1573 ± 72*	0.105
Proteins, *g/d*	95.9 ± 3.5	66.9 ± 3.4*	95.6 ± 3.4	79.3 ± 3.4*	0.012
Carbohydrates, *g /d*	187.3 ± 9.8	142.6 ± 8.1*	196.4 ± 11.5	138.9 ± 6.2*	0.579
Lipids, *g/d*	94.7 ± 4.3	69.1 ± 2.9*	108.7 ± 5.9	67.0 ± 3.8*	0.026
Fiber, *g/d*	21.4 ± 1.8	17.8 ± 1.7	22.6 ± 1.1	19.4 ± 1.2	0.748
Glycemic load	106.6 ± 8.6	72.8 ± 5.8*	112.1 ± 7.4	69.7 ± 5.1*	0.491
ω3 PUFAs , *g/d*	0.28 ± 0.01	0.28 ± 0.02	0.35 ± 0.16	0.08 ± 0.02*	0.002
Meal frequency, *meals/d*	4.6 ± 0.1	4.5 ± 0.1	5.5 ± 0.2	5.9 ± 0.2	0.100

Data are mean ± SEM, *n* = 60. *Different from baseline in each dietary group, *P* <0.05; *P* value: comparison between dietary group differences (6 months-baseline). *Abbreviations*: ω3 PUFAs, omega-3 polyunsaturated fatty acids.

**Table 2:** Anthropometric and biochemical variables in adults with MetS

				***P *** **value**		
**Variables**	**Baseline**	**2 months**	**6 months**	**Time**	**Diet**	**TimexDiet**
Weight, *kg*	102.8 ± 2.2^a^	95.4 ± 2.1^b^	94.4 ± 2.2^b^	< 0.001	0.834	0.666
BMI, *kg/m*^*2*^	36.1 ± 0.6^a^	33.5 ± 0.5^b^	33.2 ± 0.6^b^	< 0.001	0.997	0.474
WC, *cm*	114.2 ± 1.6^a^	106.8 ± 1.5^b^	105.7 ± 1.6^b^	< 0.001	0.999	0.102
DXA measurements						
Fat mass, *kg*	42.9 ± 1.2^a^	37.5 ± 1.2^b^	36.3 ± 1.2^c^	< 0.001^1^	0.469	0.432
Muscle mass, *kg*	56.6 ± 1.5^a^	54.5 ± 1.4^b^	54.8 ± 1.5^b^	< 0.001	0.694	0.239
Biochemical variables						
Cholesterol, *mg/dL*	219 ± 5^a^	197 ± 5^b^	219 ± 5^a^	< 0.001	0.568	0.751
Glucose, *mg/dL*	128 ± 5^a^	111 ± 3^b^	116 ± 4^b^	0.001^1^	0.786	0.377
TG, *mg/dL*	195 ± 13^a^	146 ± 10^b^	154 ± 12^b^	< 0.001^1^	0.730	0.955
FFA, *mmol/L*	0.56 ± 0.21^a^	0.52 ± 0.24^a,b^	0.47 ± 0.20^b^	0.016	0.122	0.461
CRP, *mg/L*	4.3 ± 0.7 ^a^	3.1 ± 0.4^a,b^	2.5 ± 0.4^b^	0.005^1^	0.597	0.183
HOMA index, *mM/L*	4.9 ± 0.4^a^	2.8 ± 0.3^b^	3.0 ± 0.4^b^	< 0.001	0.956	0.288
Leptin, *ng/mL*	21.5 ± 2.1^a^	14.5 ± 1.5^c^	17.2 ± 1.9^b^	< 0.001	0.816	0.837
Insulin, *μU/mL*	15.4 ± 1.1^a^	9.7 ± 0.9^b^	9.6 ± 1.0^b^	< 0.001^1^	0.964	0.634
BDI questionnaire	7.6 ± 0.8^a^	4.4 ± 0.6^b^	3.8 ± 0.7^c^	< 0.001	0.777	0.798
Activity level^2^	1.6 ± 0.1	1.6 ± 0.1	1.6 ± 0.1	0.154	0.697	0.184

Values are mean ± SEM *n* = 57-60. Effects of time, diet (control diet vs RESMENA diet) and time-diet interactions were analysed with repeated-measures ANOVA or Friedman test. Values in a row with different superscript letters (a, b, c) are significantly different, *P* < 0.05 by Bonferroni post hoc test, being a > b > c. *Abbreviations*: BDI, Beck Depression Inventory; BMI, body mass index; CRP, C-reactive protein; DXA, dual-energy X-ray absorptiometry; FFA, free fatty acids; MetS, Metabolic Syndrome; TG, triglycerides; WC, waist circumference.

^1^*P*-value based on non-parametric Friedman test compared the three time points of the study.

^2^Average daily exercise calculated by twenty-four physical activity questionnaire.

The comparative analysis between completers (n = 62) and dropouts (n = 25) concerning BDI score available at baseline, showed no significant differences (*P* = 0.255). Nonetheless, completers evidenced a lower mean value of BDI score at baseline (7.6 ± 0.7) in relation to those who did not finish the dietary treatment (9.3 ± 1.0).

The total number of subjects presenting a BDI score ≥10 units at baseline was 25% (9 M/6 F). After two months of weight loss treatment, this number decreased to 8.3% of the volunteers (3 M/2 F). At the end of the study (6 months later), only 6.6% of the subjects reported a BDI score ≥ 10 (2 M/2 F).

**Figure 2:**
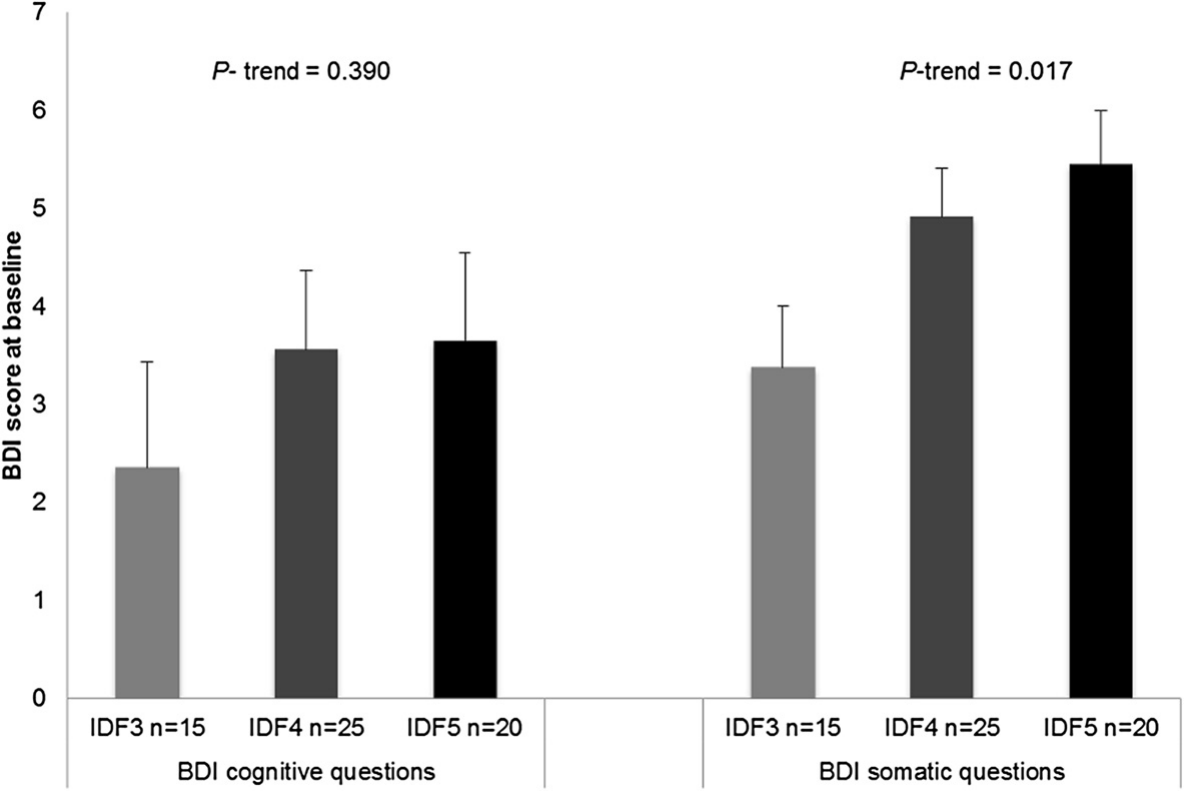
**Association between the number of MetS components assessed by IDF criteria and the BDI score at baseline.** Values are mean ± SEM, *n* = 60. BDI score divided by somatic and cognitive questions Abbreviations: BDI, Beck Depression Inventory; IDF, International Diabetes Federation.

A test of linear trend revealed that the higher the number of components of MetS according to IDF criteria (3, 4 or 5 components), the higher somatic BDI score at baseline. Interestingly, this association was not observed in the cognitive questions of the questionnaire (Figure 2).

**Figure 3:**
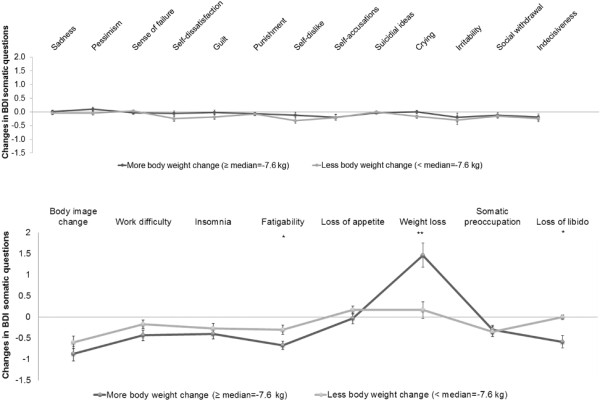
**Changes in BDI cognitive and somatic questions scores (6 months – baseline). ***n* = 60. Range from −3 to 3. Subjects with MetS were divided into more (≥median = −7.6 kg) or less (<the median = −7.6 kg) body weight change after the dietary treatment. Abbreviations: BDI, Beck Depression Inventory; MetS, Metabolic Syndrome. *p < 0.05. **p < 0.010.

In turn, losing more weight led to greater reductions (*P* < 0.013) in depressive symptoms (more weight loss ΔBDI = −5.2 ± 0.9; vs less weight loss ΔBDI = −2.5 ± 0.6). As expected, when we compared all the somatic-related questions separately between both groups (more weight loss vs less weight loss), significant differences were observed in the weight loss question, but also in the fatigability and loss of libido questions. Subjects reported losing more weight at 6 months than at baseline in the BDI questionnaire, hence the score of this question was higher at the end of the study. However, subjects who lost more weight did not show a greater reduction in the cognitive-related questions compared to individuals with lower body weight change (Figure 3).

**Figure 4:**
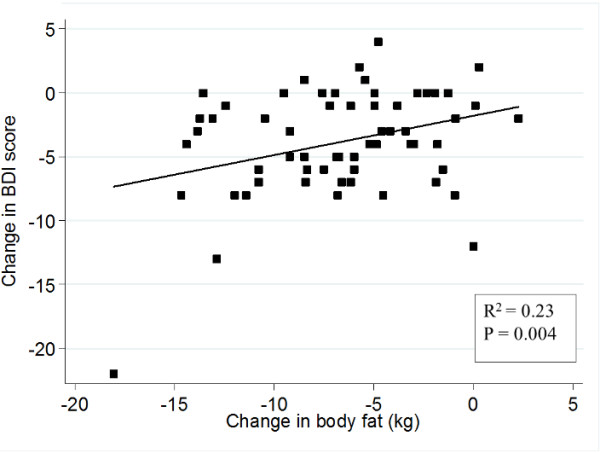
**Association between changes in BDI score and changes in body fat (kg).** Abbreviations: BDI, Beck Depression Inventory. Adjusted by sex, age and dietary group. Changes (6 months – baseline), *n* = 60.

Additionally, a positive association between changes in depressive symptoms and changes in kg of total fat mass was observed (Figure 4). We also found an association between changes in BDI score during the intervention period and changes in CRP, leptin and insulin levels. Further adjustment for body fat change eliminated the relation between depressive symptoms and insulin levels, however, the association of BDI with CRP and leptin remained statistically significant (Table 3).

**Table 3:** Association between changes in BDI score and changes in CRP, leptin and insulin

	**Change in BDI score**		
**Changes in:**	**B**	**95% CI**	**p**
CRP *mg/L*			
Unadjusted model	0.25	0.01 to 0.49	0.043
Model 1	0.27	0.03 to 0.51	0.029
Model 2	0.26	0.01 to 0.50	0.044
Model 3	0.28	0.06 to 0.51	0.015
Leptin *ng/mL*			
Unadjusted model	0.18	0.07 to 0.29	0.002
Model 1	0.21	0.10 to 0.32	0.001
Model 2	0.22	0.08 to 0.36	0.012
Model 3	0.17	0.04 to 0.30	0.013
Insulin *μU/mL*			
Unadjusted model	0.21	0.02 to 0.40	0.026
Model 1	0.25	0.07 to 0.42	0.006
Model 2	0.33	0.15 to 0.52	0.001
Model 3	0.18	−0.01 to 0.36	0.059

The table shows B coefficients (95% CI) and p-value. Changes (6 months-baseline), *n* = 60. *Abbreviation*: BDI, Beck Depression Inventory; CRP, C-reactive protein.

Model 1: Adjusted by sex, age and dietary group. Model 2: Model 1 additionally adjusted for changes in activity level. Model 3: Model 1 additionally adjusted for changes in body fat (kg).

## Discussion

The present study reports an association of the reduction in depressive manifestations with the decrease in CRP and leptin after a dietary treatment for weight loss in subjects with MetS. Moreover, the reduction in fat mass was also involved in the decrease of depressive symptoms, but it was not implicated in the association of this variable with CRP and leptin. Previous investigations have reported a decrease in depressive manifestations after a weight loss treatment [13,28], however, this study specifically demonstrates a relationship of the decrease in CRP and leptin with the reduction in self-perceived depression in subjects suffering MetS.

The domains of self-perceived depression were divided in cognitive and somatic questions in order to better interpret the primary cause of patient depression [29]. At baseline, there was a ranked relationship between increasing number of MetS components and a higher rate of somatic questions but not of cognitive components. These findings were similar to those observed in a previous study also based on self-reported BDI questionnaire [30].

Question number 19 of the BDI test, related to weight loss, was apparently distorting the total BDI score. This observation was confirmed because the weight loss was associated with the decrease in body weight at the end of the dietary treatment, showing that those subjects who lost most weight, had a higher score on the weight loss question of the test. Therefore, the weight loss question was removed from the total BDI score in some analyses. Moreover, the improvement in the fatigability and loss of libido questions (somatic-related questions) might be considered as benefits of the weight loss.

Noteworthy, a positive association between decreases in CRP values and reductions in depressive symptoms was observed as has been reported in previous studies [8]. Inflammation seems to be, in part, responsible for the link between depressive symptoms and MetS. However, the precise nature of this relationship is not clear [8]. Theoretically, the association of leptin with depression may be related both to its metabolic properties and neurobiological activities [9]. Leptin affects cognition and mood in the hippocampus, the cortex and other brain areas associated with cognition [9,31]. Obesity is related to higher levels of circulating leptin reflecting, in part, an increased formation of adipocytes, and causing leptin resistance in some obese subjects. A positive association between the decline in BDI score and the drop in leptin values was found, in line with results from previous studies [9,32]. Furthermore, in an unadjusted model, a positive association was noted between insulin and depressive symptoms, being this link well-established [33,34]. However, this association was no longer statistically significant when change in body fat was introduced in the model.

A previous weight loss intervention study showed that depression scores at baseline predicted adherence to a dietary treatment [28]. In accordance with this outcome, a higher BDI score in drop-outs at baseline was observed, although this value did not reach statistical significance. A positive association between lower BDI score and decreases in body weight was found, which is in agreement with previous studies [13]. In addition, there is some evidence suggesting that adiposity is directly related with depression [3]. In this study, the higher decrease in depressive symptoms was noticeable in those participants with a greater decline in total fat mass. Since adipose tissue is known to secrete inflammatory cytokines and leptin [35], it might be hypothesized that the decrease in body fat mass may have contributed to reduce CRP and leptin and subsequently decrease depressive symptoms.

Furthermore, the Mediterranean diet has been associated with reduced prevalence and incidence of MetS and depressive symptoms [11,12]. A healthy dietary pattern has been associated with better mood [36] and diets rich in ω3 PUFA may decrease serum cytokine production and depressive signs [37]. Also, an inverse association between both vitamin D and vitamin C levels with depressive symptoms has been observed [38,39]. In this context, we have previously shown that folate intake during this dietary intervention was well correlated with the decrease in depressive symptoms [17].

Because both MetS and depressive manifestations seem to share several common mechanisms, it has been suggested that the dietary recommendations for MetS might be helpful for depression treatment [5]. The present study strengthens this hypothesis, as the dietary treatment for MetS manifestations also reduced depressive symptoms. Moreover, several unhealthy lifestyle habits have been related with depressive symptoms, such as fast food consumption, high fat intake or low physical activity [5,40]. In this study, physical activity was not directly associated with depressive symptoms, being in agreement with previous investigations [41].

The control diet based on the AHA guidelines is a well-design strategy for weight loss [20], what may explain that no differences between dietary groups were observed in the variables analyzed in this study. As both dietary groups proved to be effective in reducing depressive symptoms and no differences between them were found, both groups were merged and analyzed together as a unique experimental group. A longitudinal observational analysis comparing the BDI scores before and after intervention was conducted, serving the volunteers as their own control. The clear differences between the beginning and the end of the study lend support to the soundness of the analysis. One of the strengths of within-subjects (paired) analyses is the reduction in error variance associated with individual differences, which increases statistical power. Moreover, in order to control the possible confounding role of the intervention group, this variable was included in the multiple-adjusted models investigating the association of anthropometric and biochemical variables with depressive manifestations in the complete sample [17].

The study has some limitations. Firstly, depressive symptoms were evaluated using a self-report questionnaire, the Beck Depression Inventory, which is not designed as a diagnostic tool but as a screening method [24]. However, this test was chosen as it is widely recognized, it has been shown to be valid for clinical assessment [25] and it has previously been used to record depressive symptoms in weight loss studies [13,28]. Secondly, this study only aimed to evaluate the association between the variables included and it cannot be determined any conclusions on causality between changes in body weight, fat mass, leptin and CRP and changes in depressive symptoms. In addition, the prevalence of depressive symptoms in our sample was low, which can be explained with the fact that subjects presenting psychiatric disorders at enrollment were not allowed to participate in the study. Also, the number of participants in this study is not very high, but it may be proposed that type-II errors were overcome since important statistical differences were found.

## Conclusions

In conclusion, this study shows an association of the reduction in depressive manifestations with CRP and leptin in subjects with MetS after following a weight loss treatment. Interestingly, the decrease in fat mass was also related with the reduction of depressive symptoms. More studies are needed to explore the mechanisms underlying the MetS-depression relationship, which may be decisive for the prevention and treatment of both conditions.

## Competing interests

The authors declared that they have no competing interests.

## Authors’ contributions

The authors contributions were as follows: APC performed the research, analysed data and wrote the manuscript; RI, PLL, IA and SNC conducted research; FL and CIL selected and contributed to the interpretation of the psychological test; MAM contributed to the statistical analysis. JAM and MAZ designed and managed the research, and had primary responsibility for final content. All the authors read and approved the final version of the manuscript.
